# A Repeat Call for the Banning of Asbestos

**DOI:** 10.1289/ehp.1002419

**Published:** 2010-07

**Authors:** Linda S. Birnbaum, Jane C. Schroeder, Hugh A. Tilson

**Affiliations:** NIEHS and NTP, National Institutes of Health, Department of Health and Human Services, Research Triangle Park, North Carolina, E-mail: birnbaumls@niehs.nih.gov; E-mail: schroederjc@niehs.nih.gov; E-mail: tilsonha@niehs.nih.gov

In 1999, the Collegium Ramazzini—an independent academic society founded to advance the study of occupational and environmental health concerns—issued a call for an international ban on the mining, manufacture, and use of asbestos (Collegium Ramazzini 1999). In the Commentary by LaDou et al. (2010) in this issue, the Collegium repeats its call for a universal ban. In our opinion, the reasons for a ban on asbestos are no less compelling now than they were 11 years go.

LaDou et al. (2010) acknowledge that asbestos has many useful properties, including a high tensile strength, the ability to be woven, and resistance to heat and most chemicals. Over the years, asbestos fibers have been used in a wide range of manufactured goods such as shingles, tiles, paper products, and textiles. Asbestos has also been widely used for thermal insulation in housing and in workplace and occupational settings. Asbestos is still used today in the manufacture of products such as asbestos-cement sheets and pipes.

LaDou et al. (2010) also note that the widespread use of asbestos products has been associated with a number of adverse health effects. More than 20 years ago asbestos was declared a proven human carcinogen by the U.S. Environmental Protection Agency (1986), the International Agency for Research on Cancer (1977), and the National Toxicology Program (1980). There is scientific consensus that exposure to asbestos causes asbestosis (a progressive fibrotic lung disease), malignant mesothelioma, and other cancers. Furthermore, there is scientific support for the position that there is no safe level of exposure to asbestos (Hillerdal 1999; Welch 2007).

World Health Assembly Resolution 58.22 on cancer prevention and control (World Health Assembly 2005) urged Member States to “… pay special attention to cancers for which avoidable exposure is a factor, particularly exposure to chemicals … in the workplace and the environment ….” In that context, the World Health Organization (WHO 2006) noted that asbestos is one of the most important occupational carcinogens and that exposure to asbestos causes approximately half of the deaths from occupational cancer. The Thirteenth Session of the joint International Labour Organization (ILO)/WHO Committee on Occupational Health (ILO/WHO 2003) recommended that special attention should be paid to asbestos-related diseases. Because of the known toxicity of asbestos, it has been banned in > 50 countries (International Ban Asbestos Secretariat 2010), and safer products have been developed to replace those containing asbestos. However, repeated efforts to ban or severely restrict chrysotile asbestos under the Rotterdam Convention—an international treaty intended to regulate global trade of dangerous chemicals—have failed (Terracini 2008). Consequently, many countries that have banned other forms of asbestos continue to allow unrestricted use of chrysotile asbestos, and chrysotile continues to be exported without the prior informed consent of importing countries [U.S. Geological Survey (USGS) 2009a].

Because there is no international ban on asbestos, the manufacture and use of asbestos products continues. The annual world production of asbestos is estimated to be > 2 million tons, and many countries having membership in the WHO continue to use asbestos (USGS 2009a). Furthermore, the use of asbestos continues in developing countries that have few resources to protect their populations. Exposure to asbestos in nonoccupational settings and in the general environment remains to be a serious health concern for public health officials. Continued use increases the likelihood of secondary exposures as older buildings are demolished during urban renewal and as asbestos-containing products are repaired or replaced (Nishikawa et al. 2008; Tse et al. 2010). It is also problematic that countries with growing economies, such as China, India, and Russia, are the largest consumers of asbestos (LaDou 2004) and that consumption increased rather than declined in China, India, and Uzbekistan between 2003 and 2007 (USGS 2009b).

According to the Collegium Ramazzini (2004), all adverse health effects and deaths associated with exposure to asbestos are preventable. The best method of prevention—banning the use of asbestos—is possible because other products developed to replace asbestos are currently in use in many countries. We agree with the Collegium that now is the time for an international ban on the mining and use of all forms of asbestos, including chrysotile asbestos. We agree that technology and “safe” work practices are not sufficient to protect workers and communities from exposure to asbestos-containing products. We also agree that all countries have a responsibility to their populations and the international community to ban all forms of asbestos.

## Figures and Tables

**Figure:**
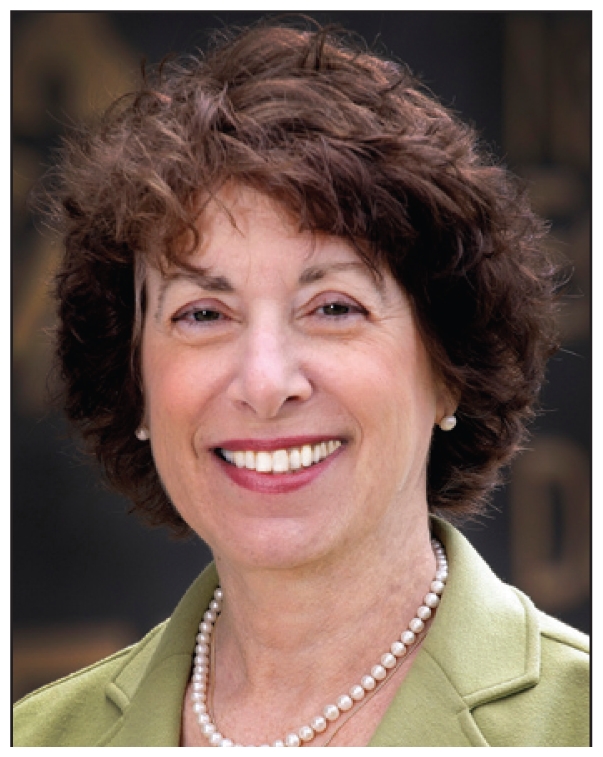
Linda S. Birnbaum

**Figure:**
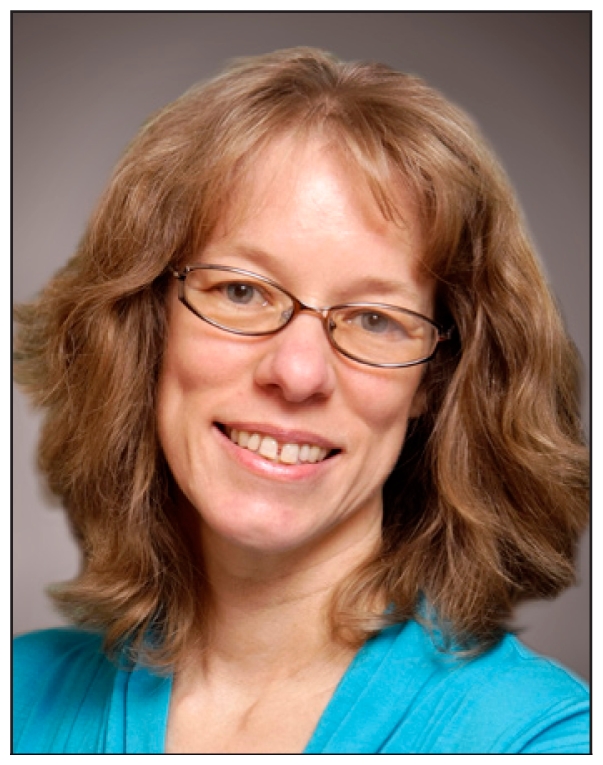
Jane C. Schroeder

**Figure:**
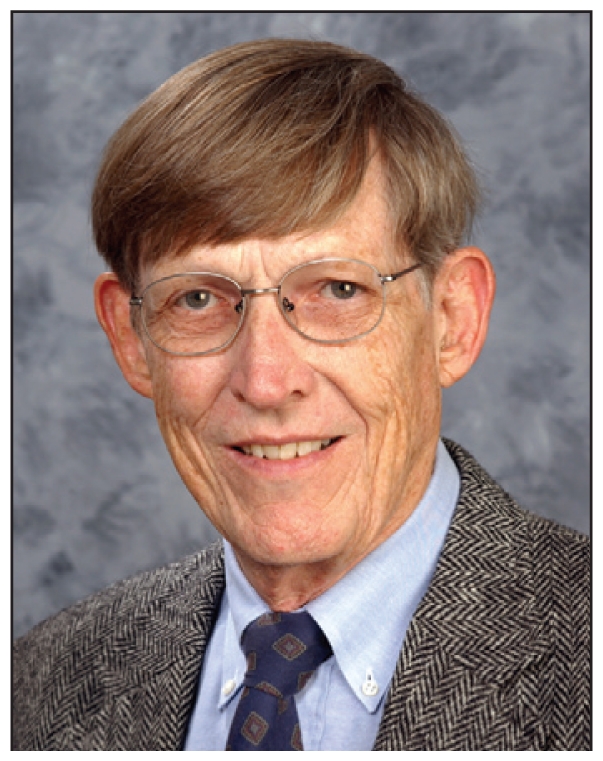
Hugh A. Tilson
